# Myhre Syndrome Misdiagnosed as Marfan Syndrome: an Educational Presentation

**DOI:** 10.21470/1678-9741-2020-0592

**Published:** 2021

**Authors:** Jinrong Li, Tao Zhu, Sufei Yang, Fan Yang, Jinhui Wu, Fei Xiong

**Affiliations:** 1 Department of Pediatrics, West China Second University Hospital, Sichuan University, Chengdu, People’s Republic of China.; 2 Key Laboratory of Birth Defects and Related Diseases of Women and Children, Sichuan University, Ministry of Education, People’s Republic of China.

**Keywords:** Growth mental deficiency syndrome of Myhre, Marfan Syndrome, Hypertension, Pulmonary, Growth Disorders, Diagnostic Errors

## Abstract

A 32-month-old girl with patent ductus arteriosus, false tendon of left ventricle, mild pulmonary hypertension, and chronic cardiac insufficiency (cardiac function level I-II) was misdiagnosed with Marfan Syndrome and there was no improvement in her physical growth after operation for this disease. The preterm baby was finally diagnosed with Myhre Syndrome by clinical phenotypes and mutation of SMAD4 gene.

**Table:** 

Abbreviations, acronyms & symbols	
**BMP** **TGF-β**	**= Bone morphogenetic protein** **= Transforming growth factor-β**

## INTRODUCTION

A 32-month-old girl was hospitalized because her growth and development were two years behind compared to same-age children. The patient was a twin (first pregnancy), and cesarean section was performed due to premature rupture of the mother’s membranes on 32 weeks of pregnancy. The birth weight of the patient was 1.4 kg, and her birth length was 34 cm. After preterm delivery, cardiac intervention and occlusion were performed when she was one year old because of patent ductus arteriosus, false tendon of left ventricle, mild pulmonary hypertension, and chronic cardiac insufficiency (cardiac function level I-II). But her physical growth was still retarded after the operation.

**Fig. 1:**
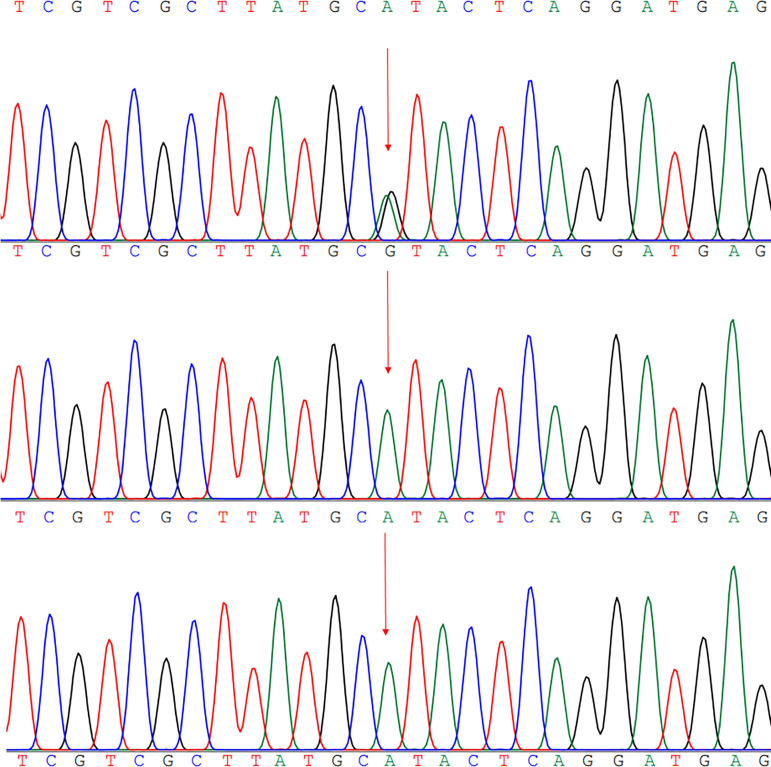
Sequencing results of the SMAD4 mutation. Gene analysis revealed a c.1498A>G of the patient (P.1500V) (a); ATP7A gene sequencing of the patient’s father was normal (b); ATP7A gene sequencing of the patient’s mother was normal (c).

Physical examination of the child showed that the clinical phenotype was not consistent with the first genetic diagnosis of Marfan syndrome. Peripheral blood samples were collected from the proband and her parents and sister in August 2018 to perform gene sequencing. The clinical phenotypes of the patient in 2018 were patent ductus arteriosus, chronic cardiac insufficiency, severe malnutrition, and stunting. Taking clinical phenotypes and gene mutations together, it was found that there was a C > T (*P*.A1152A) mutation at C3455 point on chromosome 15 (OMIM:134797), which was predicted as variant of uncertain significance by the American College of Medical Genetics and Genomics. After the operation for heart structure improvement in the following year, the child still had serious growth and development disorders, language development retardation, hearing impairment, and autism. After defining and adding new clinical phenotypes, we found that the heterozygous variation of SMAD4 gene c.1498a > G (*P*.1500v) was more reasonable. Meanwhile, the mutation of SMAD4 at this point could lead to the abnormal expression of transforming growth factor beta (TGF-β), which was a strong pathogenic, consistent with previous report (Figure 1).

QUESTIONS - What is Myhre syndrome and what is the role of SMAD4 mutation in this syndrome?
- What is the role of congenital heart disease in the differential diagnosis of Myhre syndrome?
- What is the role of gene sequencing in the diagnosis of this disease?

### Discussion of Questions

Myhre syndrome is a rare disease that affects the connective tissue. SMAD4 is located in 18q21.2, and its gene mutation is the cause of Myhre syndrome. SMAD4 gene plays an important role in the process of cell chemical signal transduction. SMAD4 can achieve signal transduction by assisting the phosphorylation and oligomerization of TGF-β and bone morphogenetic protein (BMP) pathways, and its gene mutation affects the function of multiple organ systems. The mutation of SMAD4 gene can cause four different diseases, *i.e*., juvenile polyposis syndrome, hereditary hemorrhagic telangiectasia syndrome, Myhre syndrome, and pancreatic cancers, reflecting different clinical phenotypes caused by the same gene variation ^[1]^. However, the Myhre syndrome caused by SMAD4 mutation has its own characteristics. The mutations all occur in the MH2 domain, which is necessary for SMAD4 oligomerization and TGF-β/BMP signal transduction. The restrictive pattern of SMAD4 mutation is the cause of the genetic homogeneity of Myhre syndrome, which is reflected in the clinical homogeneity ^[2,3]^. Although Myhre syndrome has a variety of symptoms and signs that affect the body's multi-organ system, patients have similar clinical phenotypes, or even if some clinical symptoms are missing in the early stage, they will gradually become more obvious with aging ^[4]^ (Question A).

Congenital heart disease is a prominent problem in patients with Myhre syndrome. It is also an important basis for clinical judgment of factors affecting growth and development. Patients with Myhre syndrome often suffer from atrial septum, ventricular septal defect, patent ductus arteriosus, and valvular stenosis at birth, and they may also present with pulmonary artery stenosis and occlusion ^[5-7]^. Heart problems may lead to poor growth. The child was born with patent ductus arteriosus, and gradually developed pulmonary hypertension and chronic cardiac insufficiency with aging. It was because of the serious heart problems that clinicians neglected the performance of other organ systems in the diagnosis of the disease, focused on the cardiovascular system in clinical practice, and got the wrong diagnosis (Question B).

In the process of gene examination, the same clinical phenotype may be caused by different gene mutations. In the analysis of a single prominent clinical phenotype, it is necessary to comprehensively summarize the clinical phenotype of the patient. When the result of gene mutation cannot explain the disease, we should carry out gene analysis from a new perspective. In this case, the gene data were analyzed for the first time only focusing on cardiovascular disease-related genes, thus making a clinical diagnosis of suspected Marfan syndrome. However, there was no catch-up growth after cardiac intervention, and the problem of growth retardation continued. At the same time, with aging, there was language retardation and autism-like behavior. Combined with the special facial features and bone and joint performance, we reinterpreted the results of gene exon sequencing, and finally detected the heterozygous variation of SMAD4 gene c.1498a > G (*P*.1500v) and corrected the clinical diagnosis (Question C).

## BRIEF CONSIDERATION OF THE CASE REPORTED

The clinical manifestations of the syndrome are various, which may show different clinical characteristics with aging. The same clinical phenotype may be caused by different gene mutations, and the same gene mutation may also lead to completely different clinical manifestations. When conducting gene analysis, we must consider it comprehensively, rather than just focus on a single system.

## LEARNING POINTS

The clinical phenotype is determined by the common factors of gene polymorphism and environment, and phenotypes of the same disease might be different at different ages.

The clinical phenotype is the basis of gene sequencing and interpretation, and the establishment of perfect genotype and clinical phenotype is an important method for the diagnosis of rare diseases.

Myhre syndrome is a multiple organ syndrome with multiple clinical manifestations.

Both gene variations of FBN1 and SMAD4 are easily misinterpreted as Marfan syndrome caused by FBN1 and misdiagnosed.

**Table:** 

Authors' roles & responsibilities	
JL	Substantial contributions to the conception or design of the work; or the acquisition, analysis, or interpretation of data for the work; drafting the work or revising it critically for important intellectual content; agreement to be accountable for all aspects of the work in ensuring that questions related to the accuracy or integrity of any part of the work are appropriately investigated and resolved; final approval of the version to be published
TZ	Substantial contributions to the conception or design of the work; or the acquisition, analysis, or interpretation of data for the work; drafting the work or revising it critically for important intellectual content; agreement to be accountable for all aspects of the work in ensuring that questions related to the accuracy or integrity of any part of the work are appropriately investigated and resolved; final approval of the version to be published
SY	Substantial contributions to the conception or design of the work; or the acquisition, analysis, or interpretation of data for the work; drafting the work or revising it critically for important intellectual content; agreement to be accountable for all aspects of the work in ensuring that questions related to the accuracy or integrity of any part of the work are appropriately investigated and resolved; final approval of the version to be published
FY	Substantial contributions to the conception or design of the work; or the acquisition, analysis, or interpretation of data for the work; drafting the work or revising it critically for important intellectual content; agreement to be accountable for all aspects of the work in ensuring that questions related to the accuracy or integrity of any part of the work are appropriately investigated and resolved; final approval of the version to be published
JW	Substantial contributions to the conception or design of the work; or the acquisition, analysis, or interpretation of data for the work; drafting the work or revising it critically for important intellectual content; agreement to be accountable for all aspects of the work in ensuring that questions related to the accuracy or integrity of any part of the work are appropriately investigated and resolved; final approval of the version to be published
FX	Substantial contributions to the conception or design of the work; or the acquisition, analysis, or interpretation of data for the work; drafting the work or revising it critically for important intellectual content; agreement to be accountable for all aspects of the work in ensuring that questions related to the accuracy or integrity of any part of the work are appropriately investigated and resolved; final approval of the version to be published
